# The role of a community health worker-delivered preconception and pregnancy intervention in achieving a more positive pregnancy experience: the *Bukhali* trial in Soweto, South Africa

**DOI:** 10.1186/s12905-024-02982-8

**Published:** 2024-03-05

**Authors:** Larske M. Soepnel, Khuthala Mabetha, Shane A. Norris, Molebogeng Motlhatlhedi, Nokuthula Nkosi, Sonja Klingberg, Stephen Lye, Catherine E. Draper

**Affiliations:** 1 SAMRC/Wits Developmental Pathways for Health Research Unit, Department of Paediatrics, Faculty of Health Sciences, School of Clinical Medicine, University of the Witwatersrand, Johannesburg, South Africa; 2 Julius Global Health, Julius Center for Health Sciences and Primary Care, University Medical Center Utrecht, Utrecht University, Utrecht, The Netherlands; 3 School of Human Development and Health, University of Southampton, Southampton, UK; 4 Lunenfeld-Tanenbaum Research Institute, Sinai Health System, Department of Obstetrics and Gynecology, Department of Physiology and Medicine, University of Toronto, Toronto, ON Canada

**Keywords:** Pregnancy experiences, Positive pregnancy experience, Postpartum, Community-health worker, Intervention, Process evaluation

## Abstract

**Background:**

A patient-centered, human-rights based approach to maternal care moves past merely reducing maternal mortality and morbidity, towards achieving a positive pregnancy experience. When evaluating an intervention, particularly in the context of the complex challenges facing maternal care in South Africa, it is therefore important to understand how intervention components are experienced by women. We aimed to qualitatively explore (i) factors influencing the pregnancy and postpartum experience amongst young women in Soweto, South Africa, and (ii) the influence of *Bukhali*, a preconception, pregnancy, and early childhood intervention delivered by community health workers (CHWs), on these experiences.

**Methods:**

Semi-structured, in-depth interviews were conducted with 15 purposively sampled participants. Participants were 18–28-year-old women who (i) were enrolled in the intervention arm of the *Bukhali* randomized controlled trial; (ii) were pregnant and delivered a child while being enrolled in the trial; and (iii) had at least one previous pregnancy prior to participation in the trial. Thematic analysis, informed by the positive pregnancy experiences framework and drawing on a codebook analysis approach, was used.

**Results:**

The themes influencing participants’ pregnancy experiences (aim 1) were participants’ feelings about being pregnant, the responsibilities of motherhood, physical and mental health challenges, unstable social support and traumatic experiences, and the pressures of socioeconomic circumstances. In terms of how support, information, and care practices influenced these factors (aim 2), four themes were generated: acceptance and mother/child bonding, growing and adapting in their role as mothers, receiving tools for their health, and having ways to cope in difficult circumstances. These processes were found to be complementary and closely linked to participant context and needs.

**Conclusion:**

Our findings suggest that, among women aged 18–28, a CHW-delivered intervention combining support, information, and care practices has the potential to positively influence women’s pregnancy experience in South Africa. In particular, emotional support and relevant information were key to better meeting participant needs. These findings can help define critical elements of CHW roles in maternal care and highlight the importance of patient-centred solutions to challenges within antenatal care.

**Trial registration:**

Pan African Clinical Trials Registry PACTR201903750173871, 27/03/2019.

**Supplementary Information:**

The online version contains supplementary material available at 10.1186/s12905-024-02982-8.

## Background

Maternal mortality is an important metric for determining progress in maternal care, as illustrated by Sustainable Development Goal target 3.1 which aims to reduce the global maternal mortality ratio to less than 70 per 100,000 live births [1, 2]. In South Africa, maternal mortality has declined steadily since 2012, as exemplified by the maternal mortality in-facility ratio, estimated at 144.9 versus 88.0 per 100,000 live births in 2012 and 2020, respectively [3]. Despite this, rates remain high compared to high-income countries, and evidence suggests that progress is hampered by socioeconomic inequalities and access to healthcare services [4, 5]. Against this background, the importance of moving beyond merely aiming for reductions in mortality and morbidity, towards a positive pregnancy experience, is increasingly acknowledged [6–8].

The 2016 World Health Organisation (WHO) “recommendations on antenatal care for a positive pregnancy experience” suggests a more woman-centred, human-rights based approach to maternal care, enabling populations to reach their full potential [6–8]. The concept of a positive pregnancy experience was defined based on a qualitative review [9] that aimed to determine what is of importance to women in pregnancy care. This review identified care practices, support, and information as three equally critical elements of antenatal care. The four main factors identified as defining a positive pregnancy experience were ‘maintaining physical and sociocultural normality’, ‘maintaining a healthy pregnancy for mother and baby – including preventing and treating risks, illness, and death’, ‘having an effective transition to positive labour and birth’, and ‘achieving positive motherhood – including maternal self-esteem, competence, and autonomy’ [9]. Similarly, 2021 WHO guidelines highlight the importance of a positive postpartum experience, by adapting to changes and maintaining or regaining health for themselves and their baby [10, 11]. In achieving these aims, and in the realm of maternal and child health more generally, the role of community health workers (CHWs) is promising, although it has been found to be dependent on context, resources, and training [12–14].

Centring pregnancy and postpartum experiences within maternal care highlights the need for data on and indicators of women’s experiences, that extend beyond, for example, the number of visits [15]. By extension, such insight is also needed when evaluating interventions. This is particularly valuable in the context of the challenges facing maternal care in South Africa, including the convergence of communicable and infectious diseases [16, 17], high rates of unintended pregnancy [18], socioeconomic inequalities [4], and gender-based violence [19].

Therefore, we aimed to evaluate the impact of a preconception, pregnancy, and early childhood intervention, *Bukhali,* on participants’ pregnancy and postpartum experiences in Soweto, South Africa. *Bukhali* is a randomised controlled trial evaluating a CHW-delivered intervention aimed at improving young women’s physical and mental health, nutrition, and health behaviours [20, 21]. Taking a life-course approach, the intervention provides continuity of care through multiple phases, including from pregnancy to postpartum. Although the socioeconomic milieu is increasingly varied, young women living in Soweto are faced with several structural and social challenges. These include experiencing traumatic events, gender-based violence, unemployment, food insecurity, mental health challenges, and limited health literacy [22–28]. There is also a high rate of unintended pregnancies in this setting, with the 2019 South African National Antenatal HIV Sentinel Survey finding that 56.5% of pregnancies amongst women aged 20–24 years old were unintended [18].Since participants are included regardless of parity, the trial provides an additional opportunity to reflect on previous pregnancies relative to the index pregnancy (during which the *Bukhali* intervention was received).

Evaluating the trial using qualitative methods helps to provide more in-depth, woman-centred understanding of participants’ perceptions of context and trial processes. Specifically, we qualitatively explored (i) factors influencing the pregnancy and postpartum experience amongst young South African women, and (ii) the influence of trial-based support, information, and care practices on these factors during a second pregnancy.

## Methods

### Study design, setting, and population

This qualitative study used semi-structured interviews to explore young women’s pregnancy experiences. The included women were participants enrolled in the intervention arm of the *Bukhali* trial in South Africa, which forms part of the global Healthy Life Trajectories Initiative (HeLTI) (Trial registration with the PAN African Clinical Trials Registry, PACTR201903750173871, Registered 27/03/2019) [21]. The trial is being conducted in the urban setting of Soweto, close to Johannesburg.

For *Bukhali,* community-based recruitment was conducted. Eligible women were aged 18–28 and living in Soweto, and were enrolled after giving informed consent. Participants were subsequently blinded and allocated to the control or intervention arm of the trial [21]. Briefly, the *Bukhali* intervention, designed to improve nutrition, physical health, and mental health in young South African women, is delivered by CHWs, known as ‘Health Helpers’ (HHs). The intervention consists of four main components: individual sessions with the HH using Healthy Conversation Skills [29] and trauma-informed care to support behaviour change; health literacy resource material; multi-micronutrient supplementation; and care services including health screening (for conditions such as high blood pressure (BP), obesity, Human Immunodeficiency Virus (HIV), and, in the preconception phase, pregnancy screening). During pregnancy, this component also includes an ultrasound scan performed by a trained radiographer. These components and the CHW-approach are outlined in Supplementary Fig. 1 ([20], reproduced with permission). Although the main outcome of the trial is offspring obesity at five years old, a phased approach is used to explore specified outcomes following each phase of the trial (preconception, pregnancy, infancy, and early childhood). *Bukhali* was designed to align with current conditions in the South African public healthcare sector; the intervention components, including during pregnancy, are aimed at enhancing existing primary health care practices [20].

We used purposive sampling of intervention-arm participants who had attended at least two sessions of the pregnancy phase, had completed the pregnancy phase (given birth), had given birth to a live child no more than 12 months previously, and had carried a previous pregnancy to term prior to participation in the trial. These participants were invited to participate in an in-depth interview regarding their pregnancy experiences, and contact attempts for data collection continued until the study had produced enough data and insights to meet the objectives of the study (*n* = 15). To achieve these 15 interviews, contact was initially attempted with 22 participants; three were not available to attend the appointment due to lack of time or not currently being in Soweto, and four were not contactable.

### Data collection

Individual in-depth interviews were conducted in-person at the *Bukhali* study site. Interviews were conducted by researchers trained in qualitative data collection (MM), with a qualitatively trained colleague taking notes (please also see the COREQ Checklist included as Supplementary File 2). Both researchers were female, familiar with the trial and the local context, and able to converse in the participants’ home languages in addition to English. The interview was conducted using a semi-structured interview guide developed by the co-authors a priori. The topics covered by the interview guide included the following: (i) information about the pregnancy and how old the baby is now; (ii) their overall experience of the pregnancy, their baby, and with *Bukhali*; (iii) how this pregnancy compared to their previous pregnancy, including comparison to standard care, their perceptions of pregnancy and motherhood, their knowledge, and health behaviours; and (v) their motivation to stay in the trial following the pregnancy. Observations and reflections about the interview were recorded as complementary notes, and all interviews were audio-recorded and subsequently transcribed *verbatim* and translated into English where necessary, by a third-party provider.

### Qualitative analysis

Thematic analysis was used, following the 6-phase approach outlined by Braun and Clarke for reflexive thematic analysis [30]. However, our methods drew on a codebook approach for thematic analysis, as this approach lends itself to the more applied, exploratory aims of this process evaluation-based study. Following familiarisation with the transcripts and field notes, LS led the development of the coding framework based on the first three transcripts, using MAXQDA version 2020 (VERBI GmbH, Berlin). During next steps of the analysis (coding, generating initial themes, developing and reviewing themes), this initial coding framework and the preliminary themes generated were discussed and modified, where required, using a ‘critical friend’ approach [31]. This included LS, KM, MM, and NN each coding the same transcript, followed by discussion, and, subsequently, the remaining transcripts being split among these same co-authors, and then merged, incorporating any additional modifications, followed by refining of the themes and writing-up of the findings by LS.

For the first objective (identifying factors influencing the participants’ pregnancy experiences, described in "Aim 1: Factors influencing pregnancy experience" section of the results), the analysis approach was more descriptive, or closer to the data [32]. An inductive approach was used to provide the flexibility to reflect these findings as participants described them and as they pertain specifically to our context. We therefore chose not to retrospectively fit these generated themes onto the four defined components of a positive pregnancy experience previously outlined by Downe et al. [9].

For the second objective (exploring in what way intervention components influence participants’ pregnancy experiences, described in "Aim 2: Trial components influencing pregnancy experiences" section of the results), the more interpretive analysis approach was informed by the concept of a positive pregnancy experience and the three related pillars of care (support, information, and care practices), as described in the 2016 WHO guidance on antenatal care for a positive pregnancy experience [6]. The ability to investigate processes (or mechanisms [33]) in an in-depth way is considered one of the strengths of qualitative methods when exploring potential causality [34]. The intervention components of ‘support’, ‘information’ and ‘care practices’ served as a conceptual framework for understanding the themes (processes through which these components influence pregnancy experiences), which were developed inductively.

## Results

Table 1 provides a disaggregated overview of participant characteristics. The results section is divided into two sections, to address each of the two aims.

**Table 1:** Participant characteristics

Variable	N (%);
**Participant age at interview**	
** 22–23**	4 (26.7)
** 24–25**	5 (33.3)
** 26–27**	2 (13.3)
** 28–29**	4 (26.7)
**Number of previous live births**	
** 1**	15 (100)
**Initiation of trial during**	
**Preconception**	7 (46.7)
**Pregnancy**	8 (53.3)
**Gestational age at first trial pregnancy session**	
** 1–3 months**	5 (33.3)
** 4–5 months**	6 (40.0)
** 6–7 months**	4 (26.7)
**Estimated time since delivery at time of interview**	
**≤ 3 months**	5 (33.3)
**3–6 months**	6 (40.0)
**6–9 months**	2 (13.3)
**9–11 months**	2 (13.3)

**Fig. 1:**
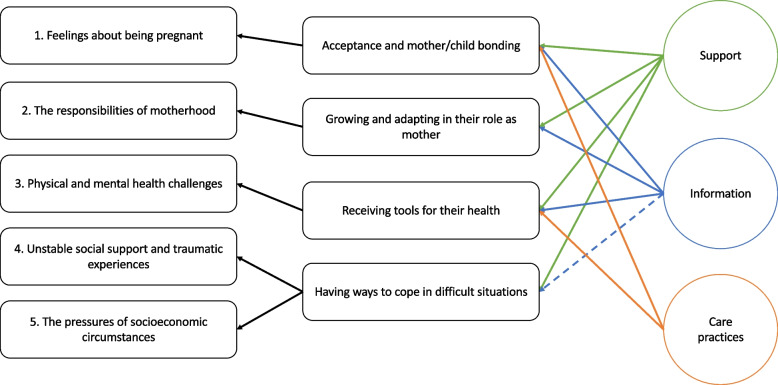
Overview of themes around factors influencing pregnancy experiences, illustrating how these relate to the three trial components (support, information, and care practices)

Figure 1 provides an overview of the factors influencing pregnancy experience (Aim 1: Factors influencing pregnancy experience) and how these relate conceptually to the three trial components. A more detailed description of the processes introduced in Fig. 1 can be found in the results section "Aim 2: Trial components influencing pregnancy experiences".

### Aim 1: Factors influencing pregnancy experience

#### Feelings about being pregnant

None of the participants described their pregnancies as being planned. Participants described feelings of shock, fear, and disappointment upon finding out about their second pregnancy. These feelings seemed to be attributable in part to participants and their families perceiving the pregnancy as negatively impacting their future prospects in terms of education and job opportunities. However, some described mixed feelings, feeling happiness and acceptance about the pregnancy despite the initial shock. Such mixed feelings seemed in part related to the reaction from their social network. Participants described fear of telling their family members about the pregnancy. Some participants additionally expressed frustration and disappointment over the sense that this was their second pregnancy. During the current pregnancy, a few participants reported that they considered opting for a termination of pregnancy, but ultimately decided to proceed with the pregnancy.

*“I started in the study while pregnant, but I didn’t know I was pregnant; like they recruited us from the street and brought us here and took blood tests and urine tests and they found that I was pregnant; so, I was shocked and all that and I didn’t want to keep the baby and I was telling [HH] that I can’t do it.” (in-depth interview [IDI] 2)*

*“I was disappointed you know; at first I was tempted to terminate because I was also not expecting it, it was still early, you know, but ja, I was happy.” (IDI 8)*

*“Yoh, I was so angry at myself because I wanted to do a lot for myself but when I got the baby, I had to put a lot of things on hold, ja, and the father of the baby was not there for me.” (IDI 10)*

#### The responsibilities of motherhood

Participants described feeling differently about motherhood with their first compared to their second child. The changes seemed to reflect an increase in maturity with older age, taking on more responsibilities with the second child, and, in some cases, feeling the burden of these formidable responsibilities more acutely. However, a number of participants also expressed feeling more bonded with their baby during the second compared to the first pregnancy and postpartum period. Being able to transfer knowledge and experience to the second postpartum period helped participants feel more comfortable and confident to manage the responsibilities of motherhood.

*“I see myself as a mother now who can care for her children properly, before I was a mother to one child right, but I didn’t feel like a mother because I still wanted to go out and party and all that stuff, but now I’m at home looking after my children and all that, ja.” (IDI 2)*

*“Like you’re not young anymore in your mind, you are no longer young, you became a mother again... and now there are two girls and like you have to be strict because I mean (sigh) it’s a lot of work raising girls these days, especially.” (IDI 1)*

*“I enjoyed the second pregnancy because I would enjoy the baby moving and would worry if they didn’t, with the second one. I didn’t care at all or notice such with the first one. I would speak to the second baby when I was pregnant and play with the baby, so it was different the first one didn’t get as much attention.” (IDI 4)*

#### Physical and mental health challenges

Participants’ experiences of health during pregnancy and delivery tended to be one of the first things they described when asked to reflect on either of their pregnancy experience, often comparing between their two pregnancies. Some of the reported physical health experiences included common pregnancy symptoms such as nausea, complications during pregnancy such as high BP, and birth complications. Participants described experiencing such complications as “tough”, in part due to the concern it raised about their child’s health. Some participants experienced more physical symptoms in their first pregnancy, but others experienced these more in their second pregnancy. In either case, this seemed to have a considerable impact on their experience of that pregnancy.

In terms of mental health, some participants described the impact of pre-existing challenges, sometimes in relation to traumatic experiences or socio-emotional situations, described in the next section, as well as those exacerbated by or arising during the pregnancy, often using the word “stress” or “distress” to describe their mental state. In reporting on how they felt during pregnancy, participants often combined their descriptions of physical and socioemotional or mental health challenges, indicating their inter-related nature. Some participants experiencing “stress” in their pregnancies felt additional worry and uncertainty over how this might impact their child’s health and development.

*“With the first pregnancy I never vomited but with the second one, I had nausea already in the first trimester, which I never had with the first.” (IDI 3)*

*“It was hard, I was always crying, sad and always sick, it was horrible, I was always tired and lonely.” (IDI 15)*

*“My pregnancy was not an easy journey, and it was a short period of time because I delivered in 7 months so I was complicated, no support system you know, pains I suffered, sickness; it was not an easy pregnancy I can say, not easy... It [having a premature birth] was painful because you don’t know what to expect; whether you are going to get the baby or not, so it was that; you don’t know what to expect.” (IDI 14)*

*“This time it was tough because I was bleeding, I was bleeding too much and with the first one I didn’t experience that; I had foetal distress only; but this one eish, it was complicated.” (IDI 8)*

#### Unstable social support and traumatic experiences

The main people providing social support to participants, including emotional and instrumental support, were the participants’ mother, the father of the child, other family members (such as the grandmother or siblings), and the father’s family. The degree of support, and from whom it came, tended to vary significantly between their first and second pregnancies, and even over the duration of the pregnancy. These variations could be attributed to changes in the participants’ circumstances, including the children having different fathers, experiencing loss and illness of family members, negative family response to the disclosure of either the first or second pregnancy, and frequent mobility between different households. Therefore, some participants experienced more social support in the first pregnancy, while others experienced more support in the second pregnancy, for example because of a new partner who was more involved. These findings seem to indicate a degree of instability and changeability in the participants’ support system and circumstances.

The social support received seemed to impact how participants experienced their pregnancies, with participants describing, for example, happiness and excitement if the baby’s father showed support during the pregnancy. On the other hand, some participants’ experiences were characterised by relationship problems or a lack of support from family or the father of the child. Notably, a number of participants also mentioned that they had no friends, with some indicating that their pregnancies negatively impacted friendships. Lastly, participants described experiencing social stressors and traumatic experiences. These included domestic violence, being forced to leave their home, and the deaths of important people in their lives, including to suicide. One participant implied, rather than explicitly stating, experiencing gender-based violence, contributing to a suicide attempt during the pregnancy.

*“Me and baby daddy we fought a lot, a lot of things went down there when I was pregnant and today sometimes I sit and cry and be like why weren’t things different,… because at the end of the day not all pregnancies are fairy tales as my first pregnancy was a fairy tale to me because I had everyone around me, but then the second one it was literally really different because it’s like everyone shifted away from me, like no sis you’re on your own.” (IDI 1)*

*“Yes, they have different fathers, but the first baby daddy is not in the picture at all, I raised my first one alone but this one the baby daddy is in the picture…It makes me really happy; I am happy.” (IDI 8)*

*“With the baby daddy, we had a lot of drama that made us end up at the police station so that damaged me a lot…I tried, I can give you a little then, I did try to kill myself in December, I was going through a lot, I don’t want to talk about it.” (IDI 4)*

#### The pressures of socioeconomic circumstances

Many participants described being financially insecure during the pregnancy and postpartum period. Aspects of this included being unemployed (and difficulty finding employment), being financially dependent on others, and not having their own place to live. For some participants, this resulted in not being the primary caregiver for (one of) their children. At the same time, as mentioned in the section "Unstable social support and traumatic experiences", becoming pregnant and having children was also felt to impact educational and employment opportunities. These circumstances during pregnancy were experienced as being “tough” and “difficult.”

*“No, I’m not working but I’m looking for a job and it’s so difficult, especially now raising two children, it’s very difficult because the income that he gets is not enough and you know families can refuse to help you sometimes and be like we don’t have go and work… I need a job, that is it, that’s all that I want; other than that everything is okay.” (IDI 1)*

*“With the first one I was not working but got work after 3 months and with the second one I was not working but things do get hard sometimes because asking for things is not nice.” (IDI 6)*

*“Well at the moment the place we are staying in is kind of small; so, there is not enough space so my first born is with my mother, ja.” (IDI 7)*

*“After I found out that I was pregnant they terminated the [work] contract…Yoh it was tough, it wasn’t nice cause I had my first born who had to go to crèche, buy her clothes, just make sure that everything is fine with her and then there’s this one on the way... at home there’s no one that works so I need to try and find food and toiletries somewhere, ja.” (IDI 11)*

### Aim 2: Trial components influencing pregnancy experiences

The components of support, information, and care practices are described separately in this section, in order to explore the specific processes by which they influence pregnancy experiences. However, there was a notable overlap between the components and the themes to which they were relevant. The processes by which these pillars impact the pregnancy experience can be categorised into four main themes, namely: acceptance and mother/child bonding; growing and adapting in their role as a mother; receiving tools for their health; and having ways to cope in difficult situations, as described in more detail below.

#### Support

Participants described a number of ways in which trial-based support impacted their pregnancy experiences. Firstly, participants indicated that receiving support, in the form of emotional support (having someone to talk to), advice, and providing guidance on coping skills seemed to help them to come to terms with their unintended pregnancies (see also Table 2 for overview).

*“I wasn’t ready for it, and it was so hard for me to accept that there’s a baby growing inside of me, but what made me excited was the support that I got from the dad and the support that [HH] was giving me, she just was like we will take it step by step and as time will go by you will understand.” (IDI 1)*

*“I did not know I was pregnant until I reached 2 months, so I was facing a lot of things … the talking, the counselling, all those things they have helped me to be cool.” (IDI 14)*

**Table 2:** Overview of processes by which the intervention impacted pregnancy experiences according to support, information, and care practices

Themes: How do components influence the pregnancy experience?				
**Components**	Acceptance and mother/child bonding	Growing and adapting in their role as mother	Receiving tools for their health	Having ways to cope in difficult situations
**Support** Emotional support, mentoring and appraisal, instrumental support within healthcare system	Participants described coming to terms with unintended pregnancy by having someone to talk to (the HHs and fellow trial participants) and receiving advice	Some participants described that mentoring, encouragement, and emotional support from HHs helped guide them in their parenting and made them feel more empowered to take on the role responsibilities of being a mother	In the case of health issues, tangible and emotional support helped participants navigate the health system	Participants reported gaining coping skills for social stressors, mental or physical health challenges, and difficult socioeconomic circumstances; Having the HH to open up to helped participants with such difficult circumstances
**Information** Physiological (health behaviours, fetal/child development, pregnancy stages); Biomedical (potential pregnancy complications, “danger signs”, and explanations about care practices); Sociocultural (how to care for the baby);	Information around pregnancy stages and childhood development helped participants look forward to and bond with their unborn baby	Participants felt more confident about caring for their child due to the information received; Participants were able to make more informed decisions about childcare and health-related choices (such as exclusive breastfeeding)	Health information served as a wake-up call, making participants more aware of the importance of their health; Information helped participants make informed decisions about their health; Participants felt reassured about their child’s health as a result of the biomedical and physiological health information received	Reading resources were used by one participant as a distraction or escape from social stressors
**Care practices** Ultrasounds, supplements, and health screening/measurements (such as for BMI or BP)	Trial ultrasounds (and learning the sex of the baby) helped participants come to terms with their pregnancy	Not applicable	Health screenings provided reassurance about participants’ and their child’s health; Participants received referrals for management in case of identified health issues; Participants attributed a number of health benefits to taking the trial supplements	Not applicable

*BMI* Body mass index, *BP* Blood pressure, *HH* Health helper, Not applicable: no direct impact found, based on data analysis and intervention design

In addition, some participants felt that HH support, mostly in the form of mentoring, advice, and encouragement, helped them grow as mothers. A few participants, on the other hand, did not report feeling a change in how they felt about motherhood.

*“As [HH] was saying the other day you’re not doing it for anybody you’re doing it for the children; and if it’s somehow going to be that you’re going to be a single parent so then okay, ja, we are there for you to advise you throughout this whole thing but somehow you as a mother you have to step up and lift yourself up and be like I’m the mom, I’m the mom here and you guys are the kids here; that was like from my understanding.” (IDI 1).*

“*Even before Bukhali I was a mother, maybe it changed a little bit, but I can’t say it has changed because of Bukhali*” *(IDI 4)*

In terms of social stressors, mental health challenges, and socioeconomic circumstances, participants seemed to receive help from HHs to find ways to cope in difficult situations. This was largely through having someone to talk to, receiving guidance and appraisal of participant circumstances, and learning new coping skills. In addition to HHs, contact with fellow trial participants was cited as another source of support in the context of challenging circumstances for some participants.

*“Now I know how to deal with my problems, whereas before, I used to think killing myself was the only option, now I know how to deal with the problems I have. I don’t stress as much…Being with [HH] and spending the time that I did with her, helped a lot… They taught us a lot about stress, that if you are stressed, you mustn’t bottle things up inside, that you should find someone you’re comfortable talking to and not keep things inside. Because the more you keep things inside, the more you hurt yourself, when you talk to someone you feel free and you can heal faster with the advice they give you. (IDI 13)”*

*“I can say they helped me with my feelings, I think I was falling more on the depression side of life, but I got better once I started coming here regularly, I saw things in brighter way, they taught me to love myself, to accept the situation that I am in, is as is.” (IDI 15)*

*“I was crying a lot with the first and second one; I was always stressed and all that… It [stress] was less when I spoke to people, yes it was less because I met another girl here at the study and we talked and all that, so it got better because I had someone to talk to.” (IDI 2)*

In addition, in the case of physical health issues, HHs provided both emotional support and instrumental support in the form of guidance through the health care system. For example, participants reported that HHs would help transport them to the clinic or attend certain visits with them. In another example of tangible support, some participants reported their HH going above and beyond in helping them with socioeconomic circumstances, including by bringing them food or other supplies.

*“You know, I was panicking for the first time I broke the water and then the blood came out, so I didn’t know what to do, because normally I don’t know that stuff I only know the pain, so I called [HH] on that day. It was midnight, so [HH] picked up the phone and she cooled me down, she talked to me, you know, she was there until I came to the hospital she stood and she was supportive for me.” (IDI 14)*

*“I actually came to my senses and even having to speak to [HH] and her supporting me you know, those kind of things like especially when I had no food I don’t know there is this church that she goes to or that her friend goes to but they would come and bring food, you know.” (IDI 12).*

Lastly, one participant reported feeling inspired by the HH to reach her goals, particularly since the HH were “peers” as young women from Soweto.

*“You get inspiration to say your peers are doing this, you know, you learn a lot about the study… so you learn to say okay you need to pick up your socks, you have to fight, you have to go to school, you have to do what you wanna do cause most of them they are my peers; I’m not comparing and I’m not jealous but they give you that thing and that hope to say you can do it, you will reach where you wanna reach, yes.” (IDI 14)*

#### Information

Participants indicated that receiving information about their pregnancy, such as the stages of pregnancy and the baby’s development, helped them to look forward to and bond with their (unborn) baby. In addition, information on how to care for the baby helped participants feel more confident and informed about the postpartum period. This finding also seems to imply that, by comparison, the knowledge or information available prior to the trial was limited for a number of participants. This was further highlighted by the reported lack of information participants felt they received during their routine ANC visits (also see Table 3 for gaps in ANC care).

*“It was mostly like when I was reading [the resource books], it was more like the excitement of a baby, once the baby arrives and then how much love you will give them once they are here; because remember as a person who is pregnant and doesn’t want the baby at first literally I love this baby throughout when like the baby was born. [Interviewer: so it was about bonding?] Yes.” (IDI 1)*

*“Sometimes I would read the book with my mother, like I said I had a difficult pregnancy because I kind of regretted falling pregnant. But they helped in terms of reading about how everything will be okay and it will work out.” (IDI 15)*

*“I am not nervous anymore, since I joined Bukhali, I have learnt a lot about raising a baby.” (IDI 5)*

**Table 3:** Identified gaps experienced in routine ANC care per support, information, and care practices

**Component**	Overview of gaps with illustrative quotations
**Support**	Reported poor treatment at these clinic visits, including rude and rushed treatment (“they always shout at you (IDI 1)”), lack of individual care, and lack of provider continuity. *“I could say that at the clinic sometimes the service that they give us is not good because they don’t know how to talk to people; like even if you have a problem there you can’t even talk to the person you’re supposed to talk to, ja, like they can’t give you the right service.” (IDI 9)* *“I can say the doctor that I got at the clinic was fine although sometimes you wouldn’t find the same doctor that was not nice, but the treatment was okay” (IDI 6)* *I’d say patient care, they don’t know how to deal with people, they are rude, they don’t care what they’re doing they just do it to get it done as long as you’re finished and gone. (IDI 2)*
**Information**	Not receiving sufficient information or explanation about pregnancy, child development, breastfeeding, or the care practices they underwent. In describing this lack of information, participants seemed to link it, not only to feeling uninformed, but also to feeling uncared for. *“At the clinic they check minor things, they don’t explain much…because at the clinic you don’t get taught much, especially about the child [growth] chart, they don’t explain the red line is for the child being overweight, the orange meaning underweight, they just wrote the information and that was that.” (IDI 3)* *“Even when you have to go to the clinic, they don’t actually tell you what is right or wrong they just give you supplements and then they just check how the baby is and that is it you know; they don’t actually check you or your mind-set and how it is, you know?” (IDI 12)* *“At* Bukhali *they give you a lot of information at the clinic they just do their job and that is that… They really don’t care at the clinic hey, if you bought in your child because they are sick, they just give the baby an injection and that is that they don’t give you all the extra information about anything.” (IDI 15)*
**Care practices**	Lack of provision of (multiple) ultrasounds; lack of personalised care and provider continuity. *And then the sonar, the different is that there you go to the sonar once; and then here you go every time you come visit, ja. (IDI 2)* *At the clinic they don’t do sonars …; like here [at* Bukhali*] they do thorough checks man; at the clinic they checked our blood because of maybe they want to push and finish with us (IDI 10)* *At the clinic I still didn’t get help even though they had the information that I got from here with my urinary infection, they couldn’t help me… I was actually quite stressed after I discovered that the doctor didn’t want to help me at the clinic; because they weren’t also sure about how bad the infection was. (IDI 7)*

For some participants, information about healthy behaviours and care practices during pregnancy and postpartum served as a “wake-up call” about the importance of their health. This allowed them to make more informed decisions around health behaviours such as diet, exercise, and breastfeeding. Meanwhile, information about the baby’s health helped put participants’ minds at ease.

*“The one thing that I learnt about was about health and how a woman should look after herself” (IDI 6)*

*“Like with my first born I didn’t breastfeed her but through the books I learnt that a baby grows stronger if you breastfeed, so those are the things that I didn’t know, ja…. My feelings have changed through this very issue that they taught me the benefits of breastfeeding, ja, my feelings have changed a lot because I now know the importance of that for the baby.” (IDI 10)*

Lastly, one participant mentioned that having the resource books to read served as a welcome distraction for social stressors.

*“Like, after reading the books I would think on it and say, oh this is how it’s supposed to be, ja. And there were some that helped me relax my mind, you find that maybe I fought with someone at home, then when I read the books I feel better.” (IDI 9)*

#### Care practices

The main care practices that participants mentioned in the interviews were the ultrasounds, supplements, and health screening/measurements (such as for BMI or BP). One of the ways in which these care practices impacted the pregnancy experience was by helping participants feel reassured and aware of their own and their baby’s health during pregnancy and postpartum.

*“They did a scan just to check if there aren’t any problems with the baby and making sure they were fine and they also making sure they check your low blood pressure if it wasn’t okay they would then refer you to a local clinic… I did appreciate the scans and see how the baby is and I like that they can even check the baby and make sure the baby is fine so I appreciate that.” (IDI 4)*

In addition, receiving ultrasounds, and specifically hearing the foetal heartbeat and learning their sex**,** helped participants come to terms with their pregnancy.

*“[Interviewer: Did you have the ultrasound services with the first baby?] No...[And with the second one?] Yes…That’s when I was like okay it’s not a joke this [the pregnancy] is real; when like the baby uh even the heart…she first said no this is a boy and then we did the second one and it’s like no this is a girl” (IDI 1)*

*“It helped me because of I was able to see the baby’s gender; usually they don’t tell us, they only tell us that the baby is fine, growing up fine, she’s not a preemie; but here I was able to see the gender which is she is a girl indeed, ja.” (IDI 8)*

For some participants, the trial care practices helped identify health problems, such as high BP, anaemia, and infections, for the first time. As a result, participants could be referred to public healthcare services for care. Participants reported facing barriers within this referral system, such as not receiving treatment or explanation following referral by the *Bukhali* team, leading to confusion and worry. Nevertheless, most participants seemed to appreciate having the health issue identified.

*“So far it has been good, it has really helped me because Bukhali was the one that discovered that my high blood is not okay… then they gave me a referral letter to go to the clinic” (IDI 6)*

*“They are quite helpful cause they have helped me with my iron in my blood was low so they have helped me a lot.” (IDI 14)*

Lastly, although a few participants reported not taking the trial supplements during pregnancy, others reported experiencing beneficial effects of the supplements in terms of feeling better or healthier in their pregnancy.

*“The supplements really helped me at that time because they boosted my appetite” (IDI 4)*

*“They [the pills] also helped me with my iron, lots of things.” (IDI 14)*

## Discussion

This study identifies ways in which young Sowetan women experience the impact of a CHW-delivered intervention during their pregnancy and postpartum period. The main factors identified as contributing to participants’ pregnancy experience were their feelings about being pregnant, the responsibilities of motherhood, physical and mental health challenges, unstable social support and traumatic experiences, and the pressures of socioeconomic circumstances. The study also highlights that trial components (support, information, and care practices) mitigated these factors through processes that facilitated ‘acceptance and mother/child bonding’, ‘growing and adapting in their role as mothers’, ‘receiving tools for their health’, and ‘having ways to cope in difficult circumstances’.

Relevant and timely information, psychosocial and emotional support, and effective care practices have been identified as key components of antenatal care [9]. Our results support their importance as three equal and complementary pillars of care, from the participants’ perspective. In fact, our results highlight that, from the participants’ perspective, the care provided by CHWs seems to be most effective when it is holistic rather than purely clinical, as has been previously suggested in ethnographic work [35]. This is highlighted by CHW's reported ability to help participants through processes involving psychosocial wellbeing (theme: ‘having ways to cope with difficult situations’) and parenthood (theme: ‘grow and adapt in their role as a mother’). The particular emphasis on the importance of information in our study likely reflects a previously identified knowledge gap around pregnancy and reproductive health among young South African women [24, 36]. However, participants’ emphasis on HH/CHW emotional support, trust, and mentorship, which aligns with other qualitative work around participants’ experiences of *Bukhali* [29, 37], indicates that these support elements may be key for effective receipt and use of information by participants.

Participant and local context are central to the identified ways in which the intervention influenced women’s pregnancy experiences, as is in line with the UK Medical Research Council (UKMRC) process evaluation framework’s emphasis on context [20, 33]. As such, the theme ‘acceptance and mother/child bonding’ was particularly relevant due to the unplanned nature of participants’ pregnancies, fear of telling their families about their pregnancy, and their often-unstable socioeconomic circumstances [37]. A better and more dynamic understanding of women’s attitudes towards their pregnancies, as opposed to simply labelling them as ‘unintended’ or unplanned, would help identify what role CHWs can play in supporting women within their social and structural realities [38]. This could include delivering preconception care information for future pregnancies to ultimately help improve participants’ autonomy in their reproductive decisions.

As part of their antenatal experience, participants also raised a number of unmet needs and gaps within current antenatal care. These included, for example, feeling rushed and judged during visits, lack of health provider continuity, long waiting times, and not receiving explanation or information. This aligns with previously identified challenges within antenatal care in South Africa that continue to form a barrier to maternal health, despite the provision of free public antenatal care [39–42]. Although the multidimensionality and complexity of these challenges will not be solved through any single approach, our results suggest that a CHW-delivered intervention such as *Bukhali* has potential to complement ANC services to better meet women’s needs. These findings, amidst an ongoing trial, require further research, including into the interventions’ integration and sustainability within existing care [43]. The outcomes of the randomised controlled trial [21] will additionally provide insights into the extent to which it impacts maternal and child health outcomes.

Our results also brought the limits of the scope of the *Bukhali* intervention, and indeed most public health initiatives, into focus. For instance, while socioeconomic circumstances, such as unemployment and financial dependence, were identified as impacting pregnancy experiences, addressing these directly falls outside the scope of the intervention. While CHW support could help women navigate such circumstances, this raises the importance of defining the (scope of the) CHW role in different settings. Similarly, intervention aspects such as the medical management of identified health issues, rely heavily on the existing health system. These considerations highlight the need for cross-disciplinary, structural support for pregnant women, alongside health interventions, which could perhaps be seen as an additional, non-clinical pillar of care that is important to the pregnancy experience in contexts such as Soweto.

This study had a number of limitations. The timing of the interviews, between 2–11 months postpartum, allowed for participants to reflect upon the entirety of the pregnancy and immediate postpartum experiences, but participants recalled their pregnancy experiences through a postpartum lens, which may have impacted their responses. Exploration of participant experiences at different stages of pregnancy could provide additional insights. In addition, the impact of the intervention on participants’ most recent pregnancy would have been difficult to tease out from changes attributable to it being their second as opposed to first pregnancy [44]. Lastly, the study formed part of a directed and applied process evaluation of the *Bukhali* trial, employing both descriptive (e.g. identified factors impacting pregnancy, which remain close to the data) as well as more explanatory, interpretive but still directed analysis [32, 45, 46]. While this is not necessarily a limitation, since the method provides insights relevant for the questions posed, it does result in less room for entirely open, interpretive qualitative analysis, which might complement our methods in exploring the complexity of pregnancy experiences [47].

## Conclusions

In conclusion, our results suggest that a CHW-delivered intervention combining support, information, and care practices has the potential to positively influence women’s pregnancy experience in South Africa. This impact is driven by interrelated processes that are closely tied to participant circumstances, priorities, and needs. The intervention particularly seemed to meet a need for emotional support and relevant information, which impacted participants in sometimes unanticipated ways, such as through acceptance of their (unintended) pregnancy. Understanding the ways in which key intervention ingredients are actually experienced by women, and in what way they are perceived to drive positive impacts, can therefore help redefine CHW program priorities and CHW roles in maternal care, to contribute to a positive pregnancy experience more effectively. In settings such as South Africa, with existing barriers to antenatal care, an overburdened health system, and socioeconomic inequalities, the identified pregnancy experiences also highlight the need for cross-disciplinary, patient-centred solutions for improving pregnancy experiences and potentially delivery outcomes.

### Supplementary Information

**Supplementary Material 1.**

**Supplementary Material 2.**

## Data Availability

The datasets used and analysed during the current study are available from the corresponding author on reasonable request.

## Abbreviations

BMI: Body mass index
BP: Blood pressure
CHW: Community health worker
HH: Health Helper
HeLTI: Healthy Life Trajectories Initiative
HIV: Human immunodeficiency virus
IDI: In-depth interview
UKMRC: UK Medical Research Council
WHO: World Health Organisation

## References

[1] World Health Organization (WHO) (2020). World health statistics 2020: monitoring health for the SDGs.

[2] THE 17 GOALS | Sustainable Development. https://sdgs.un.org/goals. Accessed 11 Apr 2023.

[3] Statistics South Africa (2022). The status of women’s health in South Africa: evidence from selected indicators Statistician-General.

[4] Bomela NJ (2020). Maternal mortality by socio-demographic characteristics and cause of death in South Africa: 2007–2015. BMC Public Health.

[5] Wabiri N, Chersich M, Shisana O, Blaauw D, Rees H, Dwane N (2016). Growing inequities in maternal health in South Africa: A comparison of serial national household surveys. BMC Pregn Childb.

[6] World Health Organization (2016). WHO recommendations on antenatal care for a positive pregnancy experience.

[7] Office of the United Nations High Commissioner for Human Rights (2010). Preventable maternal mortality and morbidity and human rights.

[8] Office of the United Nations High Commissioner for Human Rights (2020). Human rights-based approach to reduce preventable maternal morbidity and mortality: technical guidance.

[9] Downe S, Finlayson K, Tunçalp O, MetinGülmezoglu A (2016). What matters to women: a systematic scoping review to identify the processes and outcomes of antenatal care provision that are important to healthy pregnant women. BJOG An Int J Obstet Gynaecol.

[10] Finlayson K, Crossland N, Bonet M, Downe S (2020). What matters to women in the postnatal period: a meta-synthesis of qualitative studies. Plos One.

[11] World Health Organization (2022). WHO recommendations on maternal and newborn care for a positive postnatal experience.

[12] Le Roux KW, Almirol E, Rezvan PH, Le Roux IM, Mbewu N, Dippenaar E (2020). Community health workers impact on maternal and child health outcomes in rural South Africa - a non-randomized two-group comparison study. BMC Public Health.

[13] Rotheram-Borus MJ, le Roux KW, Norwood P, Katzen LS, Snyman A, le Roux I (2023). The effect of supervision on community health workers’ effectiveness with households in rural South Africa: A cluster randomized controlled trial. Plos Med.

[14] Olaniran A, Madaj B, Bar-Zev S, Van Den Broek N (2019). The roles of community health workers who provide maternal and newborn health services: case studies from Africa and Asia. BMJ Glob Heal.

[15] Lattof SR, Tuncalp Ö, Moran AC, Bucagu M, Chou D, Diaz T (2020). Developing measures for WHO recommendations on antenatal care for a positive pregnancy experience: a conceptual framework and scoping review. BMJ Open.

[16] Wong EB, Olivier SM, Gunda R, Koole O, Surujdeen A, Gareta D (2021). Convergence of infectious and non-communicable disease epidemics in rural South Africa: a cross-sectional, population-based multimorbidity study. Lancet Glob Heal.

[17] Clouse K, Motlhatlhedi M, Bonnet K, Schlundt D, Aronoff DM, Chakkalakal R (2018). “I just wish that everything is in one place”: Facilitators and barriers to continuity of care among HIV-positive, postpartum women with a non-communicable disease in South Africa. AIDS Care.

[18] Woldesenbet S, Kufa T, Lombard C, Manda S, Morof D, Cheyip M (2021). The prevalence of unintended pregnancy and its association with HIV status among pregnant women in South Africa, a national antenatal survey, 2019. Sci Rep.

[19] Mthembu J, Mabaso M, Reis S, Zuma K, Zungu N (2021). Prevalence and factors associated with intimate partner violence among the adolescent girls and young women in South Africa: findings the 2017 population based cross-sectional survey. BMC Public Health.

[20] Draper CE, Thwala N, Slemming W, Lye SJ, Norris SA (2023). Development, implementation, and process evaluation of Bukhali: an intervention from preconception to early childhood. Glob Implement Res Appl.

[21] Norris SA, Draper CE, Prioreschi A, Smuts C, Ware LJ, Dennis C (2022). Building knowledge, optimising physical and mental health and setting up healthier life trajectories in South African women ( Bukhali ): a preconception randomised control trial part of the Healthy Life Trajectories Initiative (HeLTI). BMJ Open.

[22] Kehoe SH, Wrottesley SV, Ware L, Prioreschi A, Draper C, Ward K (2021). Food insecurity, diet quality and body composition: Data from the Healthy Life Trajectories Initiative (HeLTI) pilot survey in urban Soweto. South Africa Public Health Nutr.

[23] Ware LJ, Prioreschi A, Bosire E, Cohen E, Draper CE, Lye SJ (2019). Environmental, social, and structural constraints for health behavior: perceptions of young urban black women during the preconception period—a healthy life trajectories initiative. J Nutr Educ Behav.

[24] Bosire EN, Ware LJ, Draper CE, Amato B, Kapueja L, Lye S (2021). Young women’s perceptions of life in urban south africa: Contextualising the preconception knowledge gap. Afr J Reprod Health.

[25] Redinger S, Norris SA, Pearson RM, Richter L, Rochat T (2018). First trimester antenatal depression and anxiety: prevalence and associated factors in an urban population in Soweto, South Africa. J Dev Orig Health Dis.

[26] Cohen E, Ware LJ, Prioreschi A, Draper C, Bosire E, Lye SJ (2020). Material and relational difficulties: the impact of the household environment on the emotional well-being of young black women living in Soweto South Africa. J Fam Issues.

[27] Draper CE, Bosire E, Prioreschi A, Ware LJ, Cohen E, Lye SJ (2019). Urban young women’s preferences for intervention strategies to promote physical and mental health preconception: a Healthy Life Trajectories Initiative (HeLTI). Prev Med Reports..

[28] Draper CE, Cook CJ, Redinger S, Rochat T, Prioreschi A, Rae DE (2022). Cross-sectional associations between mental health indicators and social vulnerability, with physical activity, sedentary behaviour and sleep in urban African young women. Int J Behav Nutr Phys Act.

[29] Draper CE, Mabena G, Motlhatlhedi M, Thwala N, Lawrence W, Weller S (2022). Implementation of healthy conversation skills to support behaviour change in the Bukhali trial in Soweto, South Africa: a process evaluation. SSM - Ment Heal.

[30] Braun V, Clarke V (2019). Reflecting on reflexive thematic analysis. Qual Res Sport Exerc Heal.

[31] Smith B, McGannon KR (2017). Developing rigor in qualitative research: problems and opportunities within sport and exercise psychology. Int Rev Sport Exerc Psychol.

[32] Sandelowski M, Barroso J (2003). Classifying the findings in qualitative studies. Qual Health Res.

[33] Moore GF, Audrey S, Barker M, Bond L, Bonell C, Hardeman W (2015). Process evaluation of complex interventions: medical research council guidance. BMJ.

[34] A.Maxwell J. Causality in Qualitative Research. SAGE Res Methods Found. 2019. 10.4135/9781526421036856899.

[35] Heinonen K (2021). Strengthening antenatal care towards a salutogenic approach: a meta-ethnography. Int J Environ Res Public Health.

[36] Govender D, Naidoo S, Taylor M (2019). Knowledge, attitudes and peer influences related to pregnancy, sexual and reproductive health among adolescents using maternal health services in Ugu, KwaZulu-Natal South Africa. BMC Public Health.

[37] Draper C, Motlhatlhedi M, Klingberg S, Mabetha K, Soepnel L, Pentecost M, et al. Young Women’s Health Behaviours in Context: A Qualitative Longitudinal Study in the Bukhali Trial. PsyArXiv Prepr. 2023. 10.31234/osf.io/xfhyn.

[38] Auerbach SL, Coleman-Minahan K, Alspaugh A, Aztlan EA, Stern L, Simmonds K (2023). Critiquing the unintended pregnancy framework. J Midw Womens Health.

[39] Amnesty International (2014). Struggle for maternal health: barriers to antenatal care in South Africa.

[40] Drigo L, Luvhengo M, Lebese RT, Makhado L (2020). Attitudes of pregnant women towards antenatal care services provided in primary health care facilities of Mbombela Municipality, Mpumalanga Province South Africa. Open Public Health J.

[41] Tshivhase L, Mukwevho D, Moyo I, Moloko SM (2022). Organizational related challenges in antenatal care service delivery in semi-urban healthcare facilities in Gauteng South Africa. Afr J Reprod Health.

[42] Pattinson RC, Hlongwane TMAG, Vannevel V (2019). Challenges to improve antenatal and intrapartum care in South Africa. S Afr Med J.

[43] Peters DH, Adam T, Alonge O, Agyepong IA, Tran N (2013). Implementation research: what it is and how to do it. BMJ.

[44] Nichols MR, Roux GM, Harris NR (2007). Primigravid and multigravid women: prenatal perspectives. J Perinat Educ.

[45] Finlay L (2021). Thematic analysis: : the ‘good’, the ‘bad’ and the ‘ugly’. Eur J Qual Res Psychother.

[46] Smith J, Firth J (2011). Qualitative data analysis: the framework approach. Nurse Res.

[47] Braun V, Clarke V (2022). Toward good practice in thematic analysis: Avoiding common problems and be(com)ing a knowing researcher. Int J Transgend Health.

