# Supplementary material for: The role of a community health worker-delivered preconception and pregnancy intervention in achieving a more positive pregnancy experience: the Bukhali trial in Soweto, South Africa
Source: BMC Womens Health. 2024 Mar 5;24:161. doi: 10.1186/s12905-024-02982-8 (PMC10916028; doi:10.1186/s12905-024-02982-8)

Supplementary figure 1: Overview of the community health worker approach to the *Bukhali* trial. Source: Draper CE, Thwala N, Slemming W, Lye SJ, Norris SA. Development, implementation, and process evaluation of Bukhali: an intervention from preconception to early childhood. *Glob Implement Res Appl*. 2023. doi:10.1007/s43477-023-00073-8. License: http://creativecommons.org/licenses/by/4.0/. No changes made.

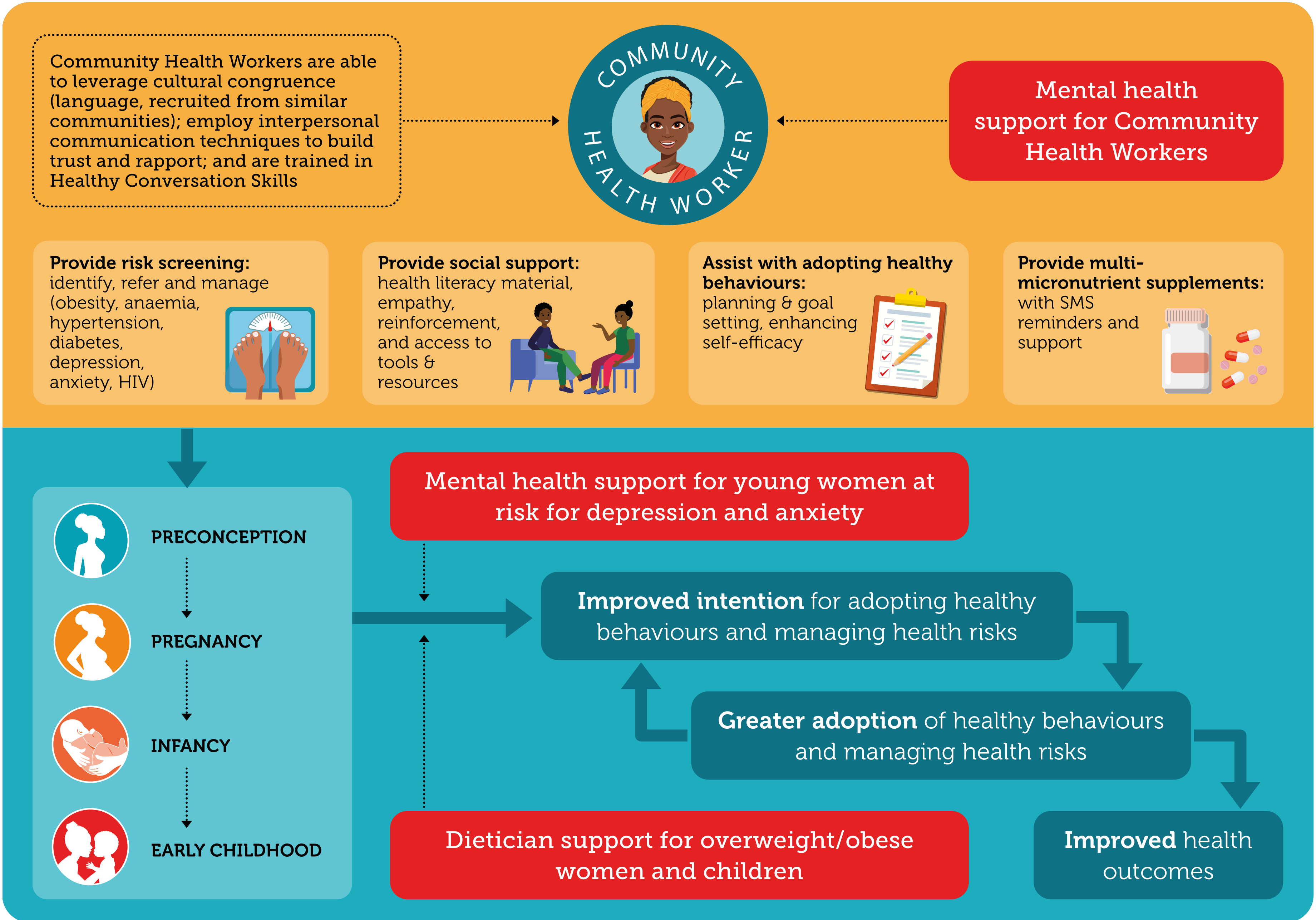

Supplement: Supplementary file 1 — Supplementary Material 1. [file 12905_2024_2982_MOESM1_ESM.pdf]
