# Supplementary material for: The role of a community health worker-delivered preconception and pregnancy intervention in achieving a more positive pregnancy experience: the Bukhali trial in Soweto, South Africa
Source: BMC Womens Health. 2024 Mar 5;24:161. doi: 10.1186/s12905-024-02982-8 (PMC10916028; doi:10.1186/s12905-024-02982-8)
Supplement: Supplementary file 2 — Supplementary Material 2. [file 12905_2024_2982_MOESM2_ESM.docx]

**Consolidated criteria for reporting qualitative studies (COREQ): 32-item checklist**

Developed from:

Tong A, Sainsbury P, Craig J. Consolidated criteria for reporting qualitative research (COREQ): a 32-item checklist for interviews and focus groups. *International Journal for Quality in Health Care*. 2007. Volume 19, Number 6: pp. 349 – 357

| **No.  Item** | **Criteria description** | **Study Information** |
| --- | --- | --- |
| **Domain 1: Research team and reﬂexivity** |  |  |
| *Personal Characteristics* |  |  |
| 1. Interviewer/ facilitator | Which author/s conducted the interview or focus group? | Molebogeng Motlhatlhedi |
| 2. Credentials | What were the researcher’s credentials? E.g. PhD, MD | BSc |
| 3. Occupation | What was their occupation at the time of the study? | Project coordinator/manager at the MRC/Wits Developmental Pathways for Health Research Unit, mentored by Catherine Draper, who has +20 years of experience in qualitative research |
| 4. Gender | Was the researcher male or female? | Female |
| 5. Experience and training | What experience or training did the researcher have? | She had 2-4 years of experiencing conducting qualitative research at the time the interviews were conducted |
| *Relationship with participants* |  |  |
| 6. Relationship established | Was a relationship established prior to study commencement? | MM, the interviewer, would have been known to a number of the participants as she had worked on the *Bukhali* trial as a study coordinator or project manager for the past 4-5 years. For those participants not acquainted with her, she would have introduced herself and her position when inviting them to the interview. |
| 7. Participant knowledge of the interviewer | What did the participants know about the researcher? e.g., personal goals, reasons for doing the research | Participants knew the interviewer’s function and job position within the *Bukhali* project. They were also informed about the reasons why the research team (all co-authors) were interested in their experiences during pregnancy, and that the data collected would be analysed and published in an academic journal. |
| 8. Interviewer characteristics | What characteristics were reported about the interviewer/facilitator? e.g., Bias, assumptions, reasons and interests in the research topic | The reasons and interests in the research topic were stated at the beginning of the focus group discussions, and the facilitators’ role in the trial was clarified. |
| **Domain 2: study design** |  |  |
| *Theoretical framework* |  |  |
| 9. Methodological orientation and Theory | What methodological orientation was stated to underpin the study? e.g. grounded theory, discourse analysis, ethnography, phenomenology, content analysis | The methodological orientation of the study was reflexive thematic analysis, informed by the positive pregnancy experiences framework and drawing on a codebook analysis approach, was used. |
| *Participant selection* |  |  |
| 10. Sampling | How were participants selected? e.g. purposive, convenience, consecutive, snowball | The participants were community health workers employed on the *Bukhali* trial; all agreed to participate in the focus groups. Community health workers are recruited using a detailed job description and routine employment procedures; a high school degree was required and previous experience in a health-related setting and the ability to relate to participants were preferred. |
| 11. Method of approach | How were participants approached? e.g. face-to-face, telephone, mail, email | Participants were approached with the help of their Health Helper, using a combination of calling, face-to-face and WhatsApp interactions to ask their interest in participating and to negotiate a convenient time to conduct the focus groups. |
| 12. Sample size | How many participants were in the study? | 15 |
| 13. Non-participation | How many people refused to participate or dropped out? Reasons? | Contact was attempted initially with 22 participants to invite them to the interviews; three of them were not available to attend the interview due to lack of time or not currently being in Soweto, and four were not contactable. |
| *Setting* |  |  |
| 14. Setting of data collection | Where was the data collected? e.g., home, clinic, workplace | At the study site at Chris Hani Baragwanath Academic Hospital |
| 15. Presence of non-participants | Was anyone else present besides the participants and researchers? | One additional team member was present as note taker during each of the interviews:  Gugulethu Mabena, female, Master of Arts, the project’s project coordinator, with 2-3 years experience in qualitative research collection |
| 16. Description of sample | What are the important characteristics of the sample? e.g., demographic data, date | Females between the ages of 22-28 years old, from the community of Soweto, Johannesburg |
| *Data collection* |  |  |
| 17. Interview guide | Were questions, prompts, guides provided by the authors? Was it pilot tested? | The semi-structured interview guide was developed collaboratively by the co-authors; some minor edits to the guide were made after debriefing the first focus group discussion. The topics covered by the interview guide included the following: (i) information about the pregnancy and how old the baby is now; (ii) their overall experience of the pregnancy, their baby, and with Bukhali; (iii) how this pregnancy compared to their previous pregnancy, including comparison to standard care, their perceptions of pregnancy and motherhood, their knowledge, and health behaviours; and (v) their motivation to stay in the trial following the pregnancy |
| 18. Repeat interviews | Were repeat interviews carried out? If yes, how many? | No |
| 19. Audio/visual recording | Did the research use audio or visual recording to collect the data? | Audio recorded |
| 20. Field notes | Were ﬁeld notes made during and/or after the interview or focus group? | Field notes were made by a designated note taker at certain points during the interview |
| 21. Duration | What was the duration of the interviews or focus group? | Between 45 and 90 minuntes. |
| 22. Data saturation | Was data saturation discussed? | Data saturation was considered when determining the number of interviews conducted. Towards the end of the 15 interviews, additional interviews were not resulting in sufficient new insights to warrant further interviews, so that it was decided within the study team to conclude data collection with the 15th interview. |
| 23. Transcripts returned | Were transcripts returned to participants for comment and/or correction? | No |
| **Domain 3: analysis and ﬁndings** |  |  |
| *Data analysis* |  |  |
| 24. Number of data coders | How many data coders coded the data? | One author (LS) led the development of the coding framework based on the first three transcripts. LS, KM, MM, and NN each coding the same transcript, followed by discussion and modification, and, subsequently, the remaining transcripts being split among these same co-authors, and then merged, incorporating any additional modifications. |
| 25. Description of the coding tree | Did authors provide a description of the coding tree? | The conceptual framework of potential themes and sub-themes was shared with co-authors for their input, before finalizing the coding framework to be applied to the transcripts. The coding framework is provided as Supplementary file 3. |
| 26. Derivation of themes | Were themes identiﬁed in advance or derived from the data? | The themes were derived from the data, but they were to some extent influenced by the pre-determined, process-evaluation-driven research questions. |
| 27. Software | What software, if applicable, was used to manage the data? | MAXQDA |
| 28. Participant checking | Did participants provide feedback on the ﬁndings? | Participants did not provide feedback on the findings. |
| *Reporting* |  |  |
| 29. Quotations presented | Were participant quotations presented to illustrate the themes/ﬁndings? Was each quotation identiﬁed? e.g. participant number | Yes. Interview number (and not the trial participant ID number) are provided, due to the risk of compromising the anonymity of the participants. |
| 30. Data and ﬁndings consistent | Was there consistency between the data presented and the ﬁndings? | Yes |
| 31. Clarity of major themes | Were major themes clearly presented in the ﬁndings? | Yes |
| 32. Clarity of minor themes | Is there a description of diverse cases or discussion of minor themes? | Yes, where these arose and within the scope of the research question. |
